# Induced membrane technique in the treatment of infected tibial bone defect: A retrospective study

**DOI:** 10.1097/MD.0000000000034280

**Published:** 2023-07-14

**Authors:** Yang Yang, Wei Zhang, Shuanji Ou, Changpeng Xu, Yong Qi, Xiangyang Ma

**Affiliations:** a The First School of Clinical Medicine, Southern Medical University, Guangzhou, China; b Department of Orthopedics, Guangdong Second Provincial General Hospital, Guangzhou, China; c Department of Orthopedics, General Hospital of Southern Theatre Command of PLA, Guangzhou, China.

**Keywords:** bone defect, cement spacer, induced membrane technique, infection, Masquelet, tibia

## Abstract

To investigate the effect of the induced membrane technique (IMT) in the treatment of infected tibial bone defect. IMT is a 2-stage procedure dedicated to reconstruction of bone defects of the limbs. Treating injuries of the tibia characterized by segmental bone loss, severe damage to the soft tissue, and a conjoining infection is a challenge using IMT. A retrospective study was performed among the patients treated using IMT for infected tibial bone defect between 2017 and 2020. The complications were recorded, and the bone defect union and the functional results were evaluated by Paley method. All patients were followed up for at least 1 year. We included 12 patients (11 males) with a mean age of 44.5 years (range 19–65). The mean length of bone defect was 26.7 mm (range 10–60). The mean interval between the stage 1 and the stage 2 of the procedure was 11.8 weeks (range 4–32). At a mean follow-up of 18.08 months (range 12–32), bone union was achieved in all cases in a mean time of 8 months (range 5–16) without infection recurrence, where 1 patient received additional bone grafting. The joint function recovered well for the patients and the rate of functionally excellent and good results was 9/12. IMT in the treatment of infected tibial bone defect offers the advantages of simple operation, use of a smaller amount of autograft bone, and low recurrence rate of infection.

## 1. Introduction

Bone defects caused by acute or secondary bone loss are injuries that rarely happen and account for <1% of all fractures.^[1]^ Local and patient-related risk factors influence the outcomes, depending on the healing and complication rates.^[2–4]^ The induced membrane technique (IMT) first described by Masquelet et al^[5]^ in 1986 has been extensively put into practice in the treatment of segmental bone defects caused by infection or tumor with a good clinical efficacy. The first stage requires radical debridement of the defect, limb stabilization, and insertion of a polymethylmethacrylate (PMMA) cement spacer into the bone defect.^[6]^ This spacer serves 2 roles. First, it inhibits the ingrowth of fibrous tissue and second, it lends the site an environment for supporting the subsequent bone graft, by its mechanical and biological functioning, respectively.^[6]^ These are achieved by eliciting a foreign body reaction that 4 to 6 weeks later results in the formation of a vascularized pseudo-periosteum, which accommodates numerous osteoprogenitor cells^[7]^ and secretes important growth factors such as vascular endothelial growth factor, transforming growth factor β1 and bone morphogenetic protein-2.^[8]^ Six to 8 weeks after the first stage, the second stage is carried out, which entails removal of the spacer without damaging the induced membrane^[7]^ and filling the defect with morcellized cancellous autologous bone graft. The bone graft is safeguarded from resorption by the pseudo-periosteum, which boosts revascularization and corticalization.^[6,8]^

It is still a challenge to treat injuries of the tibia characterized by segmental bone loss, severe damage to the soft tissue, and a conjoining infection. In particular, repeated debridement and infection have a strong impact on the soft tissue and the blood supply around the wound surface, making the treatment of chronic osteomyelitis a more difficult task. In the present retrospective study, we aimed to evaluate the outcomes of 12 patients treated in our unit using the IMT for infected bone defect of the tibia.

## 2. Materials and methods

### 2.1. Patients and study design

This single-center retrospective study was performed at the Department of Orthopedics of Guangdong Second Provincial General Hospital, which is a healthcare facility specialized in lower limb surgery. Attending surgeons performed the procedures. Eligible patients were selected by reviewing the medical records. Data such as observations, operative reports and radiographs were obtained from the medical records. The inclusion criteria were age ≥18 years and having a previous reconstruction of the infected bone defect in tibia using the IMT. Patients with defects due to bone tumor resection or with defects in bones other than the tibia, and patients whose follow-up after grafting was <12 months were excluded. The demographic data, and the data concerning the characteristics of the bone loss and the reconstruction were gathered for each patient.

All patients signed written informed consent before participation in this study and the Ethics Committee of the Guangdong Second Provincial General Hospital approved the study protocols. All the methods were performed in accordance with the relevant guidelines and regulations.

### 2.2. Surgical technique

#### 2.2.1. First stage.

The IMT is a 2-stage bone reconstruction technique. In the first stage, based on the scope of lesion determined by preoperative imaging, radical debridement was performed upon removing the internal fixation, the screw holes and the inflammatory granulation tissue in the medullary cavity, followed by the sampling of the infected tissue for the bacterial culture and the drug sensitivity test. In this practice, the sequestrum was segmentally removed until there was punctate bleeding at the broken end of the fracture. Afterwards, the fracture was fixed by external ring fixator to make sure that the length and the axis of the lower limb were in basically normal conditions. In the next steps, the antibiotic-loaded cement spacer (40 g PMMA cement mixed with 4 g vancomycin) molded as a cylinder filled the space between the 2 fracture ends, followed by drainage tube intubation process and closing the wound adequately in a tension-free manner by direct closure or use of free/local flap, if necessary. For the postoperative treatment, antibiotics were selected according to the results of bacterial culture. The second stage surgery was performed once no abnormality was observed in the reexamination of the white blood cells, C-reactive protein and erythrocyte sedimentation rate, a good wound healing was achieved locally, and no secretion in the screw hole or the sinus tract was detected.

#### 2.2.2. Second stage.

The second stage was generally performed 2 months later (Table 1). After reopening the prior incisions, the induced membrane was incised, and the spacer was removed in an atraumatic manner. The ends of each bone segment were decorticated, and the medullary canal was re-permeabilized and then cancellous bone graft was added inside the membrane. The cancellous bone graft was taken from the anterior or the posterior iliac crest. If the bone defect was very large, allogeneic cancellous bone was added. The induced membrane was then closed around the bone graft. The external ring fixator could be modified if it was found to be insufficiently functioning. Sensitive antibiotics were used for 6 weeks after the operation. In case of visible callus formation in the radiographs, patients were gradually initiated to weight-bearing exercises by crutches. The first follow-up visit was at 6 weeks postoperative. The patients were examined every 2 to 4 months until bone union, and about every 6 months afterwards.

**Table 1:** Clinical data of the patients.

Case	Gender/Age	Localization	Previous surgeries	Smoker	Fracture type	Injury type	Skin defect	Isolated microorganism	Duration with spacer (wk)	Defect length (mm)	Time to union (mo)	Functional results	Complication	Follow-up (mo)
1	M/64	Lower third	1	Yes	Closed	Car crash	No	*Staphylococcus aureus*	7	24	8	Good	Pin tract infection	12
2	M/22	Upper third	2	Yes	Closed	Fall from height	No	*Pseudomonas aeruginosa*	10	48	5	Excellent	—	32
3	M/20	Lower third	0	No	Open	Fall from height	Yes	*Enterococcus faecalis*	8	40	6	Excellent	Pin tract infection	12
4	M/47	Lower third	0	No	Open	Fall from height	Yes	*Pseudomonas aeruginosa*	32	12	16	Poor	Ankle stiffness, Delayed consolidation	29
5	F/58	Middle third	1	No	Open	Car crash	No	*Staphylococcus aureus*	16	10	8	Good	Axial deviation	24
6	M/33	Lower third	0	No	Closed	Minor trauma	No	*Staphylococcus aureus*	8	30	7	Excellent	—	12
7	M/41	Middle third	1	No	Open	Fall from height	No	*Acinetobacter baumannii*	12	20	9	Fair	Pin tract infection	14
8	M/64	Lower third	1	Yes	Open	machine crush	Yes	*Enterococcus faecalis*	4	16	10	Good	—	12
9	M/65	Middle third	1	No	Closed	Minor trauma	No	—	24	33	9	Excellent	—	13
10	M/48	Upper third	0	Yes	Closed	Pedestrian	No	*Staphylococcus aureus*	7	12	5	Excellent	Knee stiffness	23
11	M/53	Upper third	1	No	Closed	Minor trauma	No	*Pseudomonas aeruginosa*	6	15	6	Excellent	—	21
12	M/19	Upper third	3	No	Closed	Pedestrian	No	*Staphylococcus aureus*	8	60	7	Fair	Pin tract infection	13

### 2.3. Analysis

The main outcome measure was bone union. The union rate and the time to union were determined from the date of the second surgical procedure. Union was defined as the formation of a bridging callus between the graft and the host bone in 3 out of 4 cortices as observed in radiographs. The secondary outcome measures were functional results identified according to a modification of the evaluation system previously described by Paley et al.^[9,10]^ Treatment failure was defined as graft resorption and/or nonunion. Continuous variables were expressed as mean and range (minimum to maximum). Qualitative variables were expressed as proportions.

## 3. Results

### 3.1. General remarks

Twelve patients (11 males and 1 female) with a mean age of 44.5 years (range 19–65) had their infected tibia operated using the IMT between 2017 and 2020. The characteristics of the patients are to be found in Table 1. The mean length of the bone defect was 26.7 mm (range 10–60). Five patients suffered from bone exposure, 4 patients were active smokers, and most of the patients (n = 8/12) had undergone operations before they were referred to our hospital.

Infecting microorganisms were identified in samples from n = 11/12 of the patients, where the most frequently observed germs were found to be S. aureus. Also, all patients were responsive to vancomycin (Table 1). One patient was culture-negative, meaning that no microbiological growth was detected in any of the patient samples sent for culture. Nevertheless, this patient showed pus, which is an obvious sign of intra-operative infection. Also, the patient histopathologic results were consistent with leukocytosis by showing 5 neutrophils per high power field.^[11,12]^ The Infectiology Department recommended antibiotic treatment based on the antibiogram. An empirical regimen (namely, vancomycin and cefoperazone-sulbactam) was administered to the culture-negative patient. Thus, all patients were diagnosed with infected nonunion based on clinical and/or bacteriological evidence.

**Figure 1.**
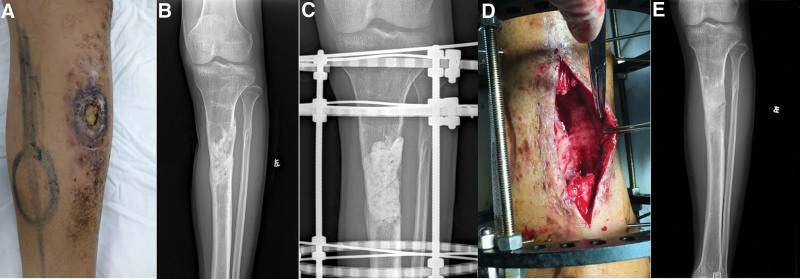
Case 2. A 22-yr-old male suffered from osteomyelitis of the left proximal tibia. (A) Preoperative photograph. (B) Preoperative radiograph. (C) After debridement, the defect was filled with antibiotic-loaded cement spacer and an external ring fixator was applied. (D) Removal of cement spacer and the retention of the induced membrane. (E) A radiograph at 2 yr follow-up showing the bone union.

**Figure 2.**
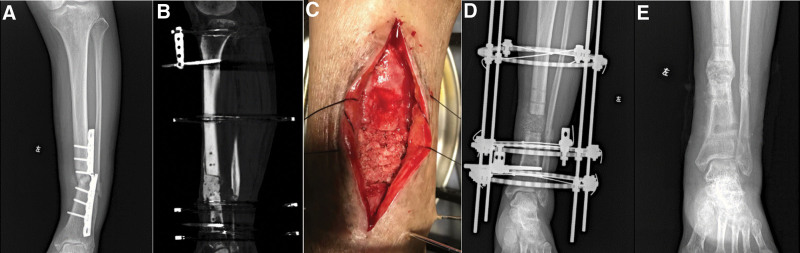
Case 8. A 64-yr-old male suffered infected nonunion and plate break of the left distal tibia. (A) Preoperative photograph. (B) The radiograph after the first stage. (C) Four weeks later the cement spacer was removed and the cancellous autografts were placed within the bone defect. (D) The radiograph after the second stage. (E) A radiograph showing the healing of the bone at 10 mo after the bone grafting.

The mean follow-up was 18.08 months (range 12–32). The follow-up was defined as the time period between completion of the second surgical stage and discharge from the clinic. The mean time from the first to the second stage operation was 11.8 weeks (range 4–32). The team of the reconstructive surgeons treated the soft tissue defects with a gastrocnemius flap in 1 and an anterolateral thigh flap in 2 patients. Upon final review, union was achieved in all 12 patients. In 11/12 patients, union was achieved without a need for further interventions. The mean time to achieve union after the second stage was 8 months (range 5–16). The functional results were graded as 6 (excellent), 3 (good), 2 (fair), and 1 (poor) (Figs. 1 and 2).

### 3.2. Complications

The criteria suggested by Paley^[13]^ were used for the assessment of the complications (Table 1). The most common complication was pin tract infection (4 patients). This was managed by proper pin tract care and oral antibiotics administered based on the bacterial culture result. Knee or ankle joint stiffness was observed in 2 patients. Physiotherapy and extending apparatus were employed for resolving this issue. One patient had axial deviation, which was managed by adjusting the frame. One patient had delayed consolidation. Supplementary iliac bone grafting was successfully used for addressing this problem. No major complication (such as nerve or vascular injury) was observed.

## 4. Discussion

Our study provides data of a homogenous set of 12 patients with infected tibia bone defects treated with the IMT. In the present study, the average interval between the stages was 11.8 weeks (the longest one was 32 weeks), where all the patients achieved good bone healing. This means that the induced membrane maintained its multipotent properties over time, where the time elapsed between the stages was only one of the factors to be accounted for bone healing. In this context, it should be pointed out that animal experiments have shown that the vascular and osteogenic activities of the induced membrane reach their peaks in 4 to 6 weeks after bone cement insertion, where the vascular activity and the osteogenic activity decrease to <60% and 40% of their peaks at the 3rd and the 2nd months, respectively.^[14,15]^ Therefore, the osteogenic effect of early bone grafting appears to be theoretically superior. Also, for noninfectious bone defects, many investigators have recommended a 4 to 8 week interval between the stages.^[16,17]^ Nevertheless, Gindraux et al have reported that the time between the first and the second stage of IMT does not have a significant impact on the speed of bone union.^[18]^ Furthermore, one should pay attention that for infected bone defects the criteria of bone grafting are difficult to be fulfilled in about 1 month.

The treatment options for infected tibial fractures are limited, necessitate adequate debridement of dead bone and elimination of necrotic tissue, and usually lead to subsequent bone defects. Some of our patients had prior multiple unsuccessful interventions, which resulted in very tenuous and compromised soft tissue envelopes that could lead to poor wound healing. Nevertheless, bone union was achieved for all of the patients in a mean time of 8 months (range 5–16), where functionally excellent and good results were obtained for 9 of them.

In the original description of the Masquelet technique, an external frame was used for fixation to avoid increasing the risk of infections due to internal fixation. The use of a locking plate as an external fixation in IMT first described by Ma et al^[19]^ is well-suited for the treatment of patients with high-impact trauma by providing a simple and comfortable treatment pathway. Thus, we employed the external ring fixator in the whole stage of IMT, where the fixator did not cross the knee or the ankle joint. This facilitated an early start for the rehabilitation process and led to an excellent knee/ankle motion and a functional recovery for the patients. Also, employing the external ring fixator allowed for the easy removal of the cement spacer in the second stage and lowered the risk of damaging the induced membrane upon filling the defect by the autologous bone graft. Note that after bone union, the removal of the external ring fixator could be carried out in a straightforward manner in an outpatient setting. The most frequent complication observed in our study was pin tract infection, which occurred due to the pin-induced skin irritation. This problem was resolved by the enhanced cleansing of the pins, their removal, and oral antibiotic therapy. Thus, no deep infection as a result of pin tract infection was developed in any of the patients. Although we did not use external ring fixator for cross-joint fixation, there were still patients who developed knee stiffness (1 patient) and ankle stiffness (1 patient). The occurrence of ankle stiffness may be related to the longer duration of external fixation, and the occurrence of knee stiffness might have been caused by a proximal external ring that was closer to the knee, thus affecting the joint flexion exercise. After bone union and the removal of external ring fixator, joint stiffness was effectively relieved in 2 patients by joint functional exercise.

Injuries of patients with tibial osteomyelitis are characterized by severe soft tissue damage and poor blood supply, which make it difficult to completely reach the local site by intravenous application of antibiotics alone. We used vancomycin as the loading antibiotic, because it is sensitive to most pathogenic bacteria, its sustained release properties are superior to those of other antibiotics, and its concentration in the local application is about 200 times that of the intravenous application.^[20]^ Local depot delivery of antibiotics increases the level of antibiotic concentration at the site multifold of the bacterial minimal inhibition concentration without increasing the antibiotic level in the blood, thus significantly reducing the systemic side effects.^[21,22]^ Hsieh et al^[23]^ have recommended that the amount of the antibiotics used in the cement should be <8 g per 40 g of the cement, because using higher amounts results in alterations in the mechanical characteristics of the bone cement. Regarding the side-effects associated with the use of vancomycin in cement, we should point out that in ref.^[24]^ for 44 patients with a total of 54 periprosthetic joint infections, the dose of the antibiotic was reported to be 4 g of vancomycin and 4.6 g of tobramycin powder per 40 g of PMMA cement, which did not result in any renal, vestibular, or hearing changes during a follow-up of at least 2 years. Furthermore, in ref.^[25]^ it has been shown that the average doses of 10.5 g vancomycin plus 12.5 g of gentamicin in antibiotic-loaded cement were clinically safe over time without any signs of systemic side effects, including acute renal insufficiency. Also, one should pay attention that although a high dose of vancomycin introduces difficulty in the molding of the bone cement and reduces its strength, in the IMT bone cement mainly plays a space-filling role and does not bear too much stress after insertion. Also, employing a high dose of vancomycin facilitates the removal of the bone cement during the second stage, which may help to protect the induced membrane more effectively. Thus, in our study we used 4 g of vancomycin per 40 g of the cement.

Morcellised cancellous bone autograft is considered as the “gold standard” of bone graft in IMT.^[6,26]^ The main source of cancellous autograft is normally the anterior or posterior iliac crest, which in many instances is followed by pain at the donor site.^[27,28]^ Cancellous allograft could partly substitute cancellous autograft,^[29]^ where the commonly-used proportion of autograft: allograft = 1:3 has rendered a union rate of ≥90%.^[30–33]^ In our study, the longest bone defect was 60 mm, and the mixed allogeneic cancellous bone constituted half of the total graft, where the follow-up revealed no bone resorption and a good bone healing. In the study of Pesciallo et al,^[34]^ the employment of the IMT and a high proportion of allograft (up to 64%) resulted in union and failure rates comparable to those reported for similar case series that used lower allograft proportions. In the second stage of bone grafting, we prepared a cigarette-like structure with an absorbable gelatin sponge and placed it on the axis of the bone graft. This was used to reduce the volume of the autogenous bone graft and simulate the hollow structure of the tibia by forming a penetrating tibial medullary cavity after bone healing. In this context, Cho et al also found that circumferential bone grafting around an absorbable gelatin sponge core decreased the amount of the grafted bone in IMT for critical-size defects of long bones and maintained the osteoinductivity of the graft with an improved vascular ingrowth.^[35]^

A summary of the relevant English literature concerning the outcome of the IMT in the treatment of tibia defects in the recent 5 years is shown in Table 2. This table gives an overview of the available literature and provides the main results of the studies, where only 2 studies were found to show bone healing rates below 50%. One of the studies that showed less successful results was presented in 2017 by Morris et al.^[51]^ In this study, 12 patients with segmental defects of the tibia were treated by IMT, where union was recorded in 5 of the 12 patients, and another 5 patients developed nonunion. The other study that showed a less satisfactory bone healing rate was that of Lotzien et al.^[36]^ In this study, bone healing within the external fixation device was detected in 8 of 31 patients (26%), and the overall healing rate including revisions was 45% (14/31). Our study would be an addition to the literature by providing an additional homogenous set of 12 patients with an infected tibia bone defect treated with the IMT. The quality of the debridement of an open fracture is a key factor that influence the infection, and hence the outcome of the technique itself. In our study, more than half of the patients underwent initial debridement at a smaller district general hospital, possibly by surgeons who were less experienced in managing these complex injuries. One patient had poor postoperative function, because for him controlling the infection took a longer time requiring performing the first stage repetitively, and weight-bearing was delayed until union had been achieved. This prolonged period of restricted weightbearing may result in other complications, such as muscle wasting and contractures, thus risking a poorer overall recovery.

**Table 2:** Papers reporting the use of IMT in the treatment of tibial defects.

Study	Total number of patients	Number of tibias (n/n or %)	Mean age (yr)	Mean defect size (cm)	Bone union rate (n/n or %)	Follow-up (mo)	Interval between stages (wk)	Time to union (mo)
Pesciallo 2021^[34]^	21	62%	40	4.2	13/13	nr	11.2	8
Lotzien 2021^[36]^	31	100%	45.8	8.3	45%	33	18.3	15.5
Deng 2020^[37]^	17	17/17	45	5.7	17/17	28.2	9.8	7.2
Baud 2020^[38]^	33	67%	39	6.7	64%	3.5	19.3	11.6
Meselhy 2020^[39]^	19	19/19	6.3	4.5	19/19	5.9	4.9	60.24
Wang G 2020^[40]^	15	15/15	39.3	6.9	15/15	20	nr	6.1
Gindraux 2020^[18]^	34	41%	45.8	67.2	11/14	nr	5.8 m	7.6
Gavaskar 2020^[41]^	26	100%	36	8.4	81%	27	6.6	5.6
Gupta S 2019^[42]^	42	38%	35	4 to 12	15/16	27.7	5.4	9
Mathieu 2019^[43]^	15	15/15	39	7.7	13/15	33	22	10.1
Raven 2019^[44]^	150	52.7%	51.4	4.4	80%	≥0%	nr	12.1
Morwood 2019^[45]^	121	46.3%	40.2	5.4	88%	22	10.7	8.8
Wang J 2019^[46]^	21	57%	37.9	5.8	12/12	19.5	12	5.5
Siboni 2018^[47]^	19	19/19	53.9	5.24	17/19	34	7.9	16
Cho 2017^[35]^	21	52%	50.2	8.9	9/11	16.4	16.2	9.1
Wu 2017^[48]^	36	53%	41.1	5.5	19/19	29.5	12.6	5.9
Tong 2017^[49]^	20	65%	39.9	6.7	13/13	25.26	9.8	nr
Qiu 2017^[50]^	22	100%	36.9	nr	73%	30	10.3	nr
Morris 2017^[51]^	12	12/12	36.8	5.8	5/12	22.5	8.2	nr
Ma 2017^[19]^	15	15/15	53.5	nr	15/15	23.4	nr	7.6
Luo F 2017^[52]^	67	100%	37	6.8	99%	22.5	nr	5.6

IMT = induced membrane technique, nr = not reported.

Our study bears certain limitations. First, this study was designed as a single-center retrospective study, which was subject to selection and indication biases. Further prospective studies are needed in the future to address methodological limitations. Second, there was only 1 cohort with 12 patients with no control group. Although a randomized, multicenter controlled trial would be ideal, performing such a trial would face ethical issues. Nevertheless, we believe the results reported in this study provide a useful insight to surgeons who wish to use this technique in their practice.

## 5. Conclusion

The IMT according to Masquelet offers an established solution for complex injuries of the tibia with segmental bone and infection. This technique has low requirements for the strength and shape of the donor bones, and its application is not influenced by the size and site of the bone defect. With new ideas proposing application of new techniques, especially in combination with bone tissue engineering, a standardized IMT treatment will lead to satisfactory clinical and radiological results in infected tibial defects.

## Author contributions

**Conceptualization:** Yong Qi, Xiangyang Ma.

**Data curation:** Wei Zhang.

**Formal analysis:** Wei Zhang.

**Investigation:** Yang Yang.

**Methodology:** Yang Yang.

**Project administration:** Yong Qi, Xiangyang Ma.

**Resources:** Changpeng Xu.

**Supervision:** Changpeng Xu.

**Writing – original draft:** Yang Yang.

**Writing – review & editing:** Shuanji Ou.
